# Grape Seed Proanthocyanidin Ameliorates LPS-induced Acute Lung Injury By Modulating M2a Macrophage Polarization *Via* the TREM2/PI3K/Akt Pathway

**DOI:** 10.1007/s10753-023-01868-5

**Published:** 2023-08-11

**Authors:** Xin Qiao, Hua Wang, Yulin He, Dongfang Song, Abdullah Altawil, Qiuyue Wang, Yan Yin

**Affiliations:** Department of Pulmonary and Critical Care Medicine, The First Hospital of China Medical University, Shenyang, China

**Keywords:** macrophage polarization, acute lung injury, grape seed proanthocyanidin, TREM2, PI3K/Akt

## Abstract

Acute lung injury (ALI) is an acute and progressive pulmonary inflammatory disease that is difficult to cure and has a poor prognosis. Macrophages, which have various phenotypes and diverse functions, play an essential role in the pathogenesis of ALI. Grape seed proanthocyanidin (GSP) has received much attention over several decades, and many biological activities such as anti-apoptotic, antioxidant, and anti-inflammatory have been identified. This study aimed to determine the effect of GSP on lipopolysaccharide (LPS)-induced ALI. In this study, we established an ALI mouse model by tracheal instillation of LPS, and by pre-injection of GSP into mice to examine the effect of GSP on the ALI mouse model. Using H&E staining, flow cytometry, and ELISA, we found that GSP attenuated LPS-induced lung pathological changes and decreased inflammatory cytokine expression in ALI mice. In addition, GSP reduced the recruitment of monocyte-derived macrophages to the lung and significantly promoted the polarization of primary mouse lung macrophages from M1 to M2a induced by LPS. *In vitro*, GSP also decreased the expression levels of inflammatory cytokines such as TNF-α, IL-6, IL-1β, and M1 macrophage marker iNOS induced by LPS in MH-S cells, while increasing the expression levels of M2a macrophage marker CD206. Bioinformatics analysis identified TREM2 and the PI3K/Akt pathway as candidate targets and signaling pathways that regulate M1/M2a macrophage polarization in ALI, respectively. Furthermore, GSP activated PI3K/Akt and increased TREM2 expression *in vivo* and *in vitro*. Meanwhile, GSP's impact on M2a polarization and inflammation suppression was attenuated by the PI3K inhibitor LY294002 or siRNA knockdown TREM2. In addition, GSP-enhanced PI3K/Akt activity was prevented by TREM2 siRNA. In conclusion, this study demonstrated that GSP could ameliorate LPS-induced ALI by modulating macrophage polarization from M1 to M2a *via* the TREM2/PI3K/Akt pathway.

**Supplementary Information:**

The online version contains supplementary material available at 10.1007/s10753-023-01868-5.

## INTRODUCTION

Acute lung injury (ALI) is a devastating respiratory disorder marked by severe hypoxemia, uncontrolled inflammatory response, and pulmonary alveolar and interstitial edema, which may progress to acute respiratory distress syndrome (ARDS) [1]. Macrophages display remarkable heterogeneity, are indispensable components of innate immunity, and play a pivotal role in inflammatory responses. There are two macrophage populations in the lungs [2]. Alveolar macrophages (AMs) are in close contact with the epithelial cells of alveoli, and interstitial macrophages (IMs) reside in the parenchyma between the microvascular endothelium and alveolar epithelium. When AMs are damaged during inflammation, circulating Ly6C^hi^ monocytes in the capillaries are recruited to the lungs and transformed into AM-like cells with diverse functions [3, 4]. Furthermore, the activation state of lung macrophages can be generally divided into two phenotypes, the classically activated (M1) phenotype and the alternatively activated (M2) phenotype [5]. M1 macrophages differentiate in response to LPS and IFN-γ produce pro-inflammatory mediators, including nitric oxide (NO), chemokines, and cytokines such as IL-1β, IL-6, IL-12, and tumor necrosis factor (TNF)-α, which promotes inflammation and increases tissue injury [6]. In contrast, M2-polarized macrophages are further classified into three subsets based on their *in vitro* responses to stimuli: M2a (characterized by the expression of CD206 receptors on the cell surface can be induced by IL-4 and IL-13), which promotes type II immune responses and fibrogenesis, M2b (characterized by the expression of CD86 receptors on the cell surface can be induced by immune complexes plus LPS) which is immunoregulatory, and M2c (characterized by the expression of CD163 and Mertk receptors on the cell surface can be induced by IL-10 and transforming growth factor-β) which is anti-inflammatory and initiates tissue remodeling [7–11]. Recent studies have demonstrated that the balance between M1/M2 macrophages is critical during the ALI process [12, 13]. In the early stage of ALI, M1 macrophages can produce inflammatory cytokines and chemokines, promote the accumulation of monocytes and neutrophils, and cause lung inflammation and tissue damage. In the rehabilitation stage of ALI, M1 macrophages become M2 macrophages and release anti-inflammatory molecules to promote inflammation regression and tissue repair. Therefore, regulation of M1/M2 macrophage polarization may be of great significance for treating ALI.

Grape seed proanthocyanidin (GSP) is an active compound extracted from grape seeds [14], and it is the source of flavone-3-ol compounds, including catechin and epicatechin monomers and their respective oligomers [15]. GSP has received much attention over several decades, and many biological activities such as anti-apoptotic, antioxidant, and anti-inflammatory have been identified [16–18]. Furthermore, it has been reported to inhibit lipid peroxidation, platelet aggregation, and tumorigenesis in tissues including skin, colon, breast, and prostate; thus it exhibited a variety of effects in treating metabolic syndrome, cancer, diabetes complications, and cardiac dysfunction after myocardial infarction [19–22]. Previous studies had been reported that GSP exerted potent anti-inflammatory activity in LPS-induced RAW264.7 macrophages by suppressing MAPK and NF-kb signal pathways [23, 24]. Nevertheless, to the best of our knowledge, whether GSP could protect against LPS-induced a mouse ALI and whether the molecular mechanism of the eventual protective effect could be associated with M2 macrophage subtype differentiation have yet to be thoroughly studied. Therefore, we systematically studied the molecular mechanism of the anti-ALI effects of GSP: 1. We established that GSP could inhibit ALI progression and promoted the polarization of macrophages from M1 to M2 in a mouse model of ALI. 2. We used bioinformatics to screen the M2a polarization-related genes and pathways as candidate targets for treating ALI. 3. *In vivo* and *in vitro* experiments verified the mechanism of action of GSP against ALI. A workflow of this study is shown in Supplementary material (Fig. S1).

## MATERIALS AND METHODS

### Acquisition and Analysis of Datasets

The microarray data of GSE61298 were downloaded from the Gene Expression Omnibus (http://www.ncbi.nlm.nih.gov/geo/) database, and the platform was GPL15207 Affymetrix Human Gene Expression Array, which included seven different macrophage subtypes in three biological replicates. In this study, replicated 1–3 in M-CSF-differentiated macrophages activated with 20 ng/ml IL-4 were selected as the M2a-polarized macrophage group. And replicated 1–3 in M-CSF-differentiated macrophages activated with 100 ng/ml LPS + 25 ng/ml IFN-γ were selected as the M1-polarized macrophage group.

### Screening of Differentially Expressed-related Genes (DEGs) of Macrophage Polarization Associated With ALI

The normalized expression matrix of microarray data was downloaded from the GSE61298 dataset. Then the probe was annotated with the annotation files from the dataset. The repeatability of data in GSE61298 was verified by principal component analysis (PCA). The GEO2R online tool was used to identify the differentially expressed polarization-related genes (M2a vs. M1). Genes with an adjusted *P*-value < 0.05 and absolute fold-change (FC) value > 1.0 were considered DEGs. A volcano map was drawn of the DEGs. GeneCards (https://www.genecards.org/) is a comprehensive database of functions involving proteomics, genomics, and transcriptomics [25]. The keywords “ALI” and “ARDS” were utilized to screen the ALI-associated targets. Genes with a relevance score greater than ten were selected as candidate ALI targets. We combined the top 600 DEGs with ALI-associated target genes to obtain the overlapping genes, used for subsequent analysis. The Venn diagram was displayed to take the intersection of the DEGs and the ALI targets through the online website (http://jvenn.toulouse.inra.fr/app/example.html). The heat map, Kyoto Encyclopedia of Genes and Genomes (KEGG) pathway, and Gene Ontology (GO) analysis, including the biological process (BP), cellular component (CC), and molecular function (MF) of the intersection genes, were conducted with the online website (https://www.bioinformatics.com.cn/).

### Molecular Docking Verification of the Binding of the GSP to the Target Protein

3D protein structures of targets were downloaded from RCSB Protein Data Bank (RCSB PDB, http://www.pdb.org/). AutoDock Tools 1.5.6 software was used to remove the water molecules, add the nonpolar hydrogen and calculate Gasteiger charges for the structure and save it as a PDBQT file. The 3D structure of the GSP was downloaded from the PubChem database (https://pubchem.ncbi.nlm.nih.gov/), then using Open Babel GUI software to convert the SDF format into PDB format. We adopted Autodock tools 1.5.6 software for docking procedures, and calculated the molecular binding energy. Next, the output molecular docking results were imported into PyMOL and Discovery Studio 2016 software for molecular docking verification display [26].

### Reagents and Antibodies

Grape seed proanthocyanidin, purchased from Macklin (#G832674, Shanghai, China) with a purity greater than 95%, was dissolved in dimethyl sulfoxide (DMSO, Sigma, #D5879) at a concentration of 100 mg/ml as a stock solution, and further diluted in phosphate buffer saline (PBS) when administrated. Lipopolysaccharide (LPS) (Escherichia coli O55:B5, #L2880) was purchased from Sigma-Aldrich (St. Louis, MO, USA). Anti-TREM2 (E6T1P)(#61788), anti-β-actin (D6A8)(#8457), and anti-rabbit IgG HRP-linked (#7074) antibodies were purchased from Cell Signaling Technology (Beverly, MA, USA). Anti-CD206 (#ab64693), anti-iNOS (#ab178945), and anti-PI3K/AKT signaling pathway (#ab283852) antibodies were purchased from Abcam (Cambridge, UK). Anti-iNOS (PE-conjugated, #696806), anti-CD206 (MMR, APC-conjugated, #141708), anti-CD86 (APC-conjugated, #105012), anti-CD163 (APC-conjugated, #155306), anti-CD11c (PerCP-conjugated, #117326), anti-Ly6C (BV605-conjugated, #128036), and anti-CD11b (FITC-conjugated, #101205) antibodies were purchased from BioLegend (San Diego, CA, USA). LY294002 (#S1105) was purchased from SelleckChem (Houston, Texas, USA).

### Murine Model of LPS‑induced ALI and Drug Treatment

Male C57BL/6 mice (6 to 8 weeks of age, 18–22 g) were purchased from the HuaFuKang company (Beijing, China). The animals were housed in groups of up to fifteen per cage with a 12/12 h light/dark cycle, adequate temperature (23 ± 2 °C), full accessed to food and water, and environmental enrichment. Animal care and experiments were performed by the Guide for the Care and Use of Laboratory Animals of the National Institutes of Health (NIH publications NO.8023, revised 1978). The Ethics Committee for Animal Use and Care of China Medical University approved all animal experiments (CMU2023662). As shown in Fig. S2, all mice were randomly divided into seven groups (20 mice every group): Control group, LPS group, 50 mg/kg GSP group, LPS + 25 mg/kg GSP group, LPS + 50 mg/kg GSP group, LPS + 75 mg/kg GSP group and LPS + 75 mg/kg GSP + 10 mg/kg LY294002 group. The mice were intraperitoneally injected with different doses of GSP (diluted in PBS) for two consecutive days. LY294002 (0.2 mg/mice) was intraperitoneally injected following the final GSP injection in the LPS + GSP + LY294002 group. At one hour after the last GSP treatment, the mice were anesthetized, and then LPS (10 mg/kg) or PBS was injected into the trachea. After LPS administration, the mice were placed vertically position and slowly shaken for one min to ensure LPS or PBS was distributed evenly between the left and right lungs. Mice were sacrificed at 24 h post LPS stimulation. The whole lungs (n = 12) were lavaged with 0.5 mL normal saline for the first time and 0.8 ml normal saline for the following four times. The recovery rate of the fluid was approximately 90%. Cells in bronchoalveolar lavage fluid (BALF) were collected by centrifugation (300 g for 10 min at 4 ◦C) for cell counts, macrophage isolation, and flow cytometry assay, and the supernatant of the first BALF was prepared for ELISA assay. The left lungs of 6–8 mice from each group were inflated intratracheally with 4% paraformaldehyde, followed by fixed in 4% paraformaldehyde for 48 h and embedded with paraffin for histology and immunohistochemistry.

### Flow Cytometry Analysis

The BALF was centrifugated at 300 g for 10 min, and the pellets were resuspended in a staining buffer (BD Biosciences, San Diego, CA, USA). 1 × 10^6^ cells were added in tubes for each sample. After that, the cells were incubated with FcR Blocking Reagent (Miltenyi, Auburn, CA, USA) to increase the specificity of the antibody. A third of the cells were first stained with anti-CD11b (macrophage surface marker), anti-CD11c (alveolar macrophage surface marker), and anti-Ly6C (monocyte surface marker) antibodies, fixed and permeabilized with fixation/permeabilization buffer (BD Biosciences, San Diego, CA, USA) and then stained with the anti-CD206 (M2a marker) plus anti-iNOS (M1 marker) antibodies. The remaining cells were first stained with anti-CD11b plus anti-CD86 (M2b marker) or anti-CD163 (M2c marker) antibodies, fixed and permeabilized with fixation/permeabilization buffer and then stained with the anti-iNOS antibody. The data were acquired on BD Celesta instrument (BD Bioscience), and macrophages were gated by CD11b^+^ with at least 3,000 events gated and analyzed with FlowJo software (Tree Star, Ashland, OR, USA).

### Measurement of Pro-inflammatory Cytokines in BALF or Cell Culture Supernatants

The BALF supernatant was collected and stored at −80 °C. Cells were cultured under the indicated conditions, and culture supernatants were collected. Then the pro-inflammatory cytokines TNF-α, IL-6, and IL-1β levels were determined in BALF and cell culture supernatants *via* ELISA kits (Boster, Wuhan, China).

### BALF Analysis

The BALF was collected, and the red blood cells were lysed using lysis buffer (Solarbio, Beijing, China), followed by centrifugation. The remaining cells were washed, resuspended with PBS, and counted using a hemocytometer. The supernatant was used for protein concentration assay using the bicinchoninic acid (BCA) protein assay kit (Beyotime, Shanghai, China) according to the manufacturer’s instructions.

### Lung Wet/dry (W/D) Weight Measurement

The W/D ratio assessed the severity of pulmonary edema. Briefly, after the mice sacrifice, the right upper lobe of the lung was removed and immediately weighed for the wet weight (W). Then the wet lung tissues were placed in an oven at 65 °C for at least 48 h until the weight no longer changed and weighed again to obtain the dry weight (D). Next, we calculated the W/D ratio.

### Lung Histopathology

After fixation with 4% paraformaldehyde for 48 h, the lung tissues were paraffin-embedded according to the standard protocol. Subsequently, the paraffin blocks were sectioned at 4um thickness and stained with hematoxylin and eosin (H&E) before a microscope histological exam. Finally, pathologists blinded to the treatment group performed the lung histopathology and injury score analysis. The severity of lung damage was scored based on the following histologic features, as described previously [27]: alveolar congestion, hemorrhage, infiltration or aggregation of neutrophils in airspace or vessel wall, and thickness of alveolar wall/hyaline membrane formation. Each item was graded on a five-point scale from 0 to 4: 0 (normal: no injury), 1 (mild damage: injury to 25% of the field), 2 (moderate damage: injury to 50% of the field), 3 (severe damage: injury to 75% of the field), and 4 (very severe damage: diffuse injury), respectively. The four variables were summed to represent the lung injury score (total score, 0–16).

### Immunohistochemical Analysis

The presence of phosphorylated phosphoinositide 3-kinase (*p*-PI3K) and phosphorylated protein kinase B (*p*-Akt) was determined in paraffin sections by immunohistochemical staining according to Histostain-Plus Kit (MXB, Fujian, China). Slices of lung tissue were taken off paraffin by dimethylbenzene and alcohol washes. Lung tissue was incubated with primary antibody of TREM2 (ab245227) at 1:150 (Abcam, Cambridge, UK), *p*-PI3K (ab182651) at 1:150 (Abcam, Cambridge, UK), and *p*-Akt (ab192623) at 1:100 (Abcam, Cambridge, UK) overnight at 4 ◦C. The next day, the lung tissue sections were washed with PBS and incubated with HRP-conjugated secondary antibody for 10 min, followed by the incubation of streptomycin anti-biotin protein-peroxidase for 10 min at room temperature. Finally, positive cells with brown color were visualized using a DAB substrate solution (MXB, Fujian, China), and the sections were counterstained with hematoxylin. The results were observed by a light microscope (Olympus BX51, Japan) at × 400 magnification. The cell with TREM2 or *p*-PI3K, or* p*-Akt immunopositivity was counted visually in three radom fields.

### Cell Culture and Intervention

MH-S cells, the SV40 transformed mouse alveolar macrophage cell line (CRI-2019, ATCC, Baltimore, Md, USA), were grown in RPMI-1640 medium (Gibco, CA, USA) supplemented with 10% fetal bovine serum (Gibco, Australia Origin) and 1% penicillin/streptomycin. Cells were cultured at 37 °C in a 5% CO_2_ and a 95% air-humidified incubator. MH-S cells were activated M1 macrophages with the indicated stimulants (according to previous studies): 1 μg/mL LPS (#L2880, Sigma, Mo) 3 h for M1 polarization [27]. The GSP (50 or 100 μg/ml) was added 24 h before LPS stimulation. We constructed TREM2 knockdown cells with siRNA or cells pretreated with LY294002 (20 μM) 2 h before LPS stimulation to explore the potential molecular mechanism. The cell culture and interfering conditions were displayed in the Fig. S3. All experiments were repeated at least three times.

### Cell Viability Assay

MH-S cells were seeded into 96-well plates (5 × 10^3^ cells/well) and treated with various concentrations of GSP for 24 h. The medium was then removed and replaced with 100ul of serum-free medium per well, and 10ul CCK8 reagent (DOJINDO, Japan) was added to each well. Next, the OD value at 450 nm was measured 1 h later to access cell viability.

### Reverse Transcription and Real-time PCR (RT-PCR)

The BALF was collected, and the red blood cells were lysed using lysis buffer (Solarbio, Beijing, China), followed by centrifugation. The pellets got from BALF were resuspended using RMPI 1640. After culturing in 6 well plates for 2 h, the adherent cells were regarded as lung macrophages for further experiments [28]. Total RNA was extracted from primary lung macrophages and MH-S cells using RNAiso Plus reagent (Takara, Dalian, China). Following standard protocols, isolated RNA was reverse-transcribed into cDNA using a Reverse Transcription Kit (Takara, Dalian, China). The RT-PCR primers are listed in Table 1. RT-PCR was performed using a qPCR kit (Takara, Dalian, China) on Roche 480 LightCycler (Basel, Switzerland). Specifically, the final reaction system was initial denaturation at 95 °C for 30 s, followed by 40 cycles of 95 °C for 5 s and 60 °C for 30 s. Gene expression was quantified using the 2 ^−ΔΔ^ comparative critical threshold method and normalized to the expression of β-actin.

**Table 1:** Primers Used for Reverse Transcription-Quantitative PCR

**mRNA**	**Primers**	**Sequences (5′–3′)**
TREM2	Upstream Downstream	CTGGAACCGTCACCATCACTC CGAAACTCGATGACTCCTCGG
IL-1β	Upstream Downstream	GAAATGCCACCTTTTGACAGTG TGGATGCTCTCATCAGGACAG
TNF-α	Upstream Downstream	ACCACGCTCTTCTGTCTACTG ATGATCTGAGTGTGAGGGTCTG
IL-6	Upstream Downstream	GTCGGAGGCTTAATTACACATGTTC GCAAGTGCATCATCGTTGTTCA
iNOS	Upstream Downstream	CAGGAAGAAATGCAGGAGATGG TGTCCTGAACGTAGACCTTGG
CD206	Upstream Downstream	TCCATTACAACCAAAGCTGACC CCTATCACAATCAGGAGGACCA
CD86	Upstream Downstream	GTGTGTTCTGGAAACGGAGTC GATGAGCAGCATCACAAGGAG
Mertk	Upstream Downstream	TGCCACCTGCACAGTGAGAA GGTTGACGAGGGTGCGTAATC
β-actin	Upstream Downstream	CATCCGTAAAGACCTCTATGCCAAC ATGGAGCCACCGATCCACA

### Western Blotting

MH-S cells were lysed in the mixture of RIPA lysis buffer (Beyotime, Shanghai, China) and Protein phosphatase inhibitor (Beyotime, Shanghai, China). Cell lysates were centrifuged at 12,000 rpm for 30 min. The total protein concentration of the supernatants was assessed with a BCA kit (Beyotime, Shanghai, China). Equal amounts of the extracted proteins were separated on 8%-10% SDS–polyacrylamide gels and then transferred onto PVDF membranes. The membranes were blocked with 5% BSA or 5% fat-free milk for 2 h and then incubated with diluted primary antibodies, including anti-PI3K (1:1000 dilution), anti-*p*-PI3K (1:500 dilution), anti-Akt (1:000 dilution), anti-*p*-Akt (1:1000 dilution), anti-TREM2 (1:1000 dilution), anti-iNOS (1:1000 dilution), anti-CD206 (1:1000 dilution), and anti-β-actin (1:1000 dilution) overnight at 4 °C. Then, the membranes were washed with 0.1% Tween-20 in Tris-buffered saline (TBS) and incubated with secondary antibodies (1:2000–1:5000) for 1 h at room temperature with gentle shaking. An ECL Plus Western Blotting Detection kit (Beyotime, Shanghai, China) performed the densitometric quantification of antibody-specific bands. We used the Bio-Rad imaging system for exposure and photography. The gray values of the bands were measured by Image J software.

### Knockdown of TREM2 in MH-S Cells By siRNA

MH-S cells cultured in 6-well plates were transfected with 50 nM siRNAs (Sangon Biotech, Shanghai, China) target TREM2 (5’-AGAUGCUGGGCACCAACUUCATT-3’) and a control siRNA (5’-UUCUCCGAACGUGUCACGUTT-3’) when they reached 50% confluence. Lipofectamine 2000 transfection reagent (Invitrogen, Camarillo, CA, USA) and the siRNAs were premixed in OPTI medium (Invitrogen, Camarillo, CA, USA) according to the manufacturer’s instructions and then applied to the cells. After 24 h of transfection, the OPTI-medium was replaced by RPMI-1640 medium with 10% FBS and 1% penicillin/streptomycin. Then, MH-S cells were pretreated with GSP for 24 h, followed by treatment with LPS (1 μg/mL) for another 3 h.

### Statistical Analysis

The data are expressed as the means ± SD. Statistical significance was determined using GraphPad Prism 8.0.2 software (San Diego, CA, USA). A Student’s t-test was used for comparisons of two groups. A *p* < 0.05 was required for the results to be considered statistically significant.

## RESULTS

### GSP Exerts Protective Effects Against LPS-induced ALI *In Vivo*

**Fig. 1:**
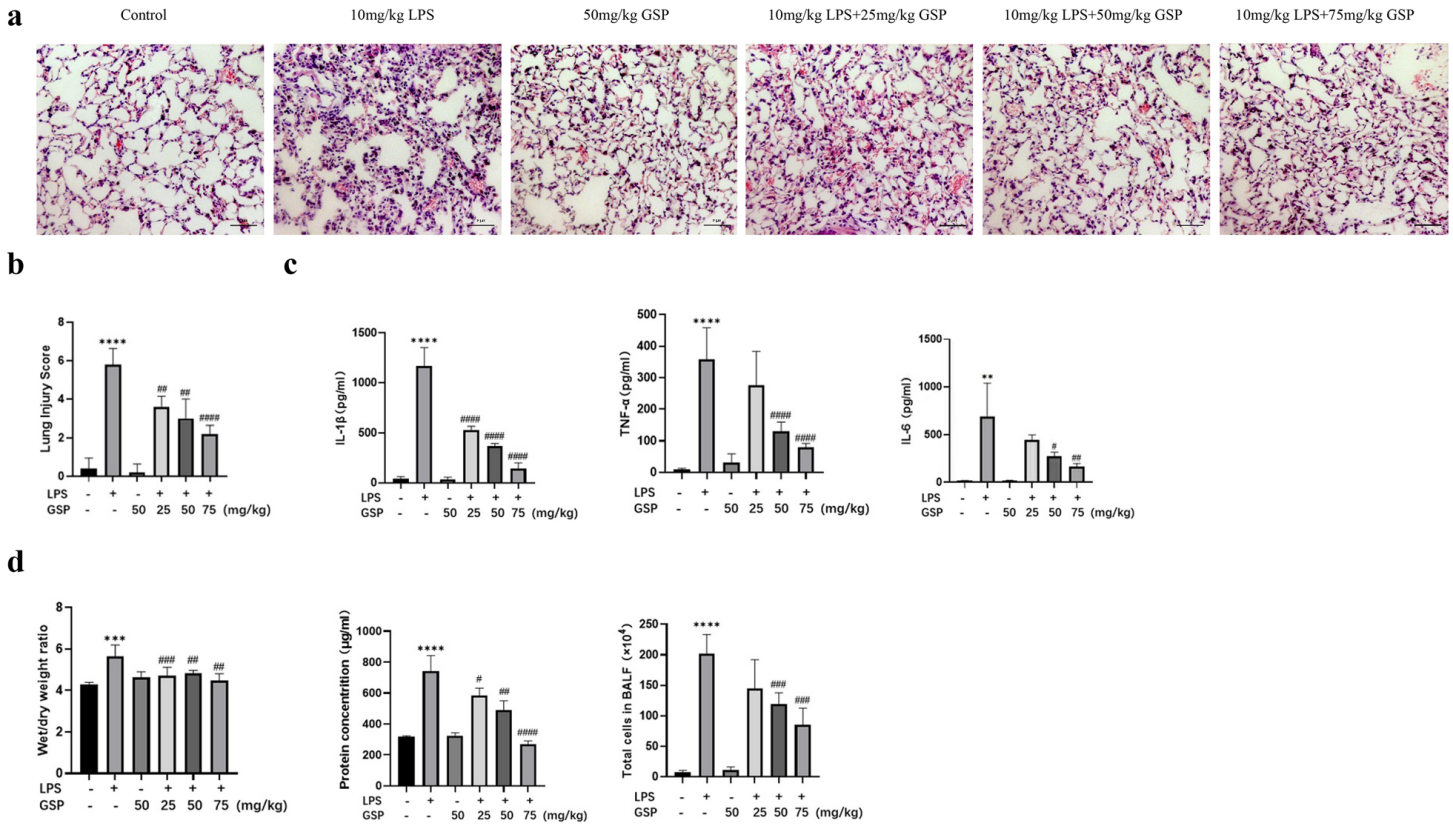
GSP attenuated LPS-induced acute lung injury. **a** Sections of lung tissues were stained with H&E (magnification 200 ×). **b** Lung injury score analysis. **c** IL-1β, TNF-α and IL-6 levels in BALF. **d** Lung wet/dry (W/D) weight ratio, the total protein concentration in the supernatant, and the total cell count in BALF after red blood cell lysis. The values represented the means ± SD. ^**^ *p* < 0.01, ^***^ *p* < 0.001, ^****^ *p* < 0.0001. vs. the control group; ^#^ *p* < 0.05, ^##^ *p* < 0.01, ^###^ *p* < 0.001, ^####^ *p* < 0.0001 vs. the LPS group, n = 5/group.

Histopathological analysis showed that the LPS challenge induced distinct histological changes compared to tissue from naive animals, including alveolar congestion, interstitial edema, alveolar wall thickening, and a mass of inflammatory cells infiltrating the lung alveolar spaces. GSP treatment significantly alleviated these LPS-induced histopathological changes (Fig. 1A). As shown in Fig. 1B, GSP (25, 50, and 75 mg/kg) significantly alleviated LPS-induced lung injury scores. We evaluated inflammatory response, pulmonary edema, and lung alveolar permeability based on pro-inflammatory cytokines, lung W/D weight ratio, protein concentration, and total cell counts in BALF. As expected, treatment with GSP (50 and 75 mg/kg) decreased the inflammatory cytokines levels (IL-1β, TNF-α, and IL-6), lung edema, total protein concentration, and the cell counts (Fig. 1C, D) in BALF. These results showed that GSP protected against LPS-induced lung injury in mice.

### GSP Reduced the Recruitment of Monocyte-derived Macrophages to the Lung

**Fig. 2:**
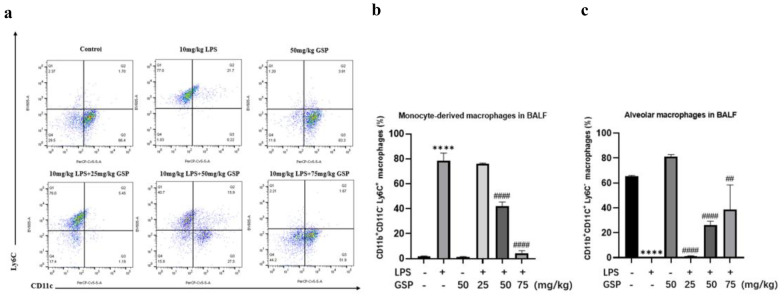
The effect of GSP on macrophage subpopulations after LPS administration. **a**-**c** Flow cytometry analyses of the percentage of CD11b^+^CD11c^−^Ly6C^+^ monocyte-derived macrophages and CD11b^+^CD11c^+^Ly6C^−^ alveolar macrophages in BALF of mice. The results were the means ± SD. ****p < 0.0001 vs. the Control group; ^##^*p* < 0.01, ^###^*p* < 0.001, ^####^*p* < 0.0001 vs. the LPS group.

As shown in Fig. 2A, we identified macrophage subpopulations according to flow cytometry analysis of cell surface protein markers under LPS stimulation without GSP pretreatment. We found that the percentage of monocyte-derived macrophages (CD11b^+^CD11c^−^Ly6C^+^) in mice BALF was significantly increased, and the percentage of alveolar macrophages (CD11b^+^CD11c^+^Ly6C^−^) was decreased after LPS administration. However, GSP (50 and 75 mg/kg) injection reduced the percentage of monocyte-derived macrophages while increasing the percentage of alveolar macrophages in the lungs of mice subjected to LPS (Fig. 2B, C).

### GSP Promoted Macrophage Shift M1 to M2a Subtype *In Vivo* and *In Vitro*

**Fig. 3:**
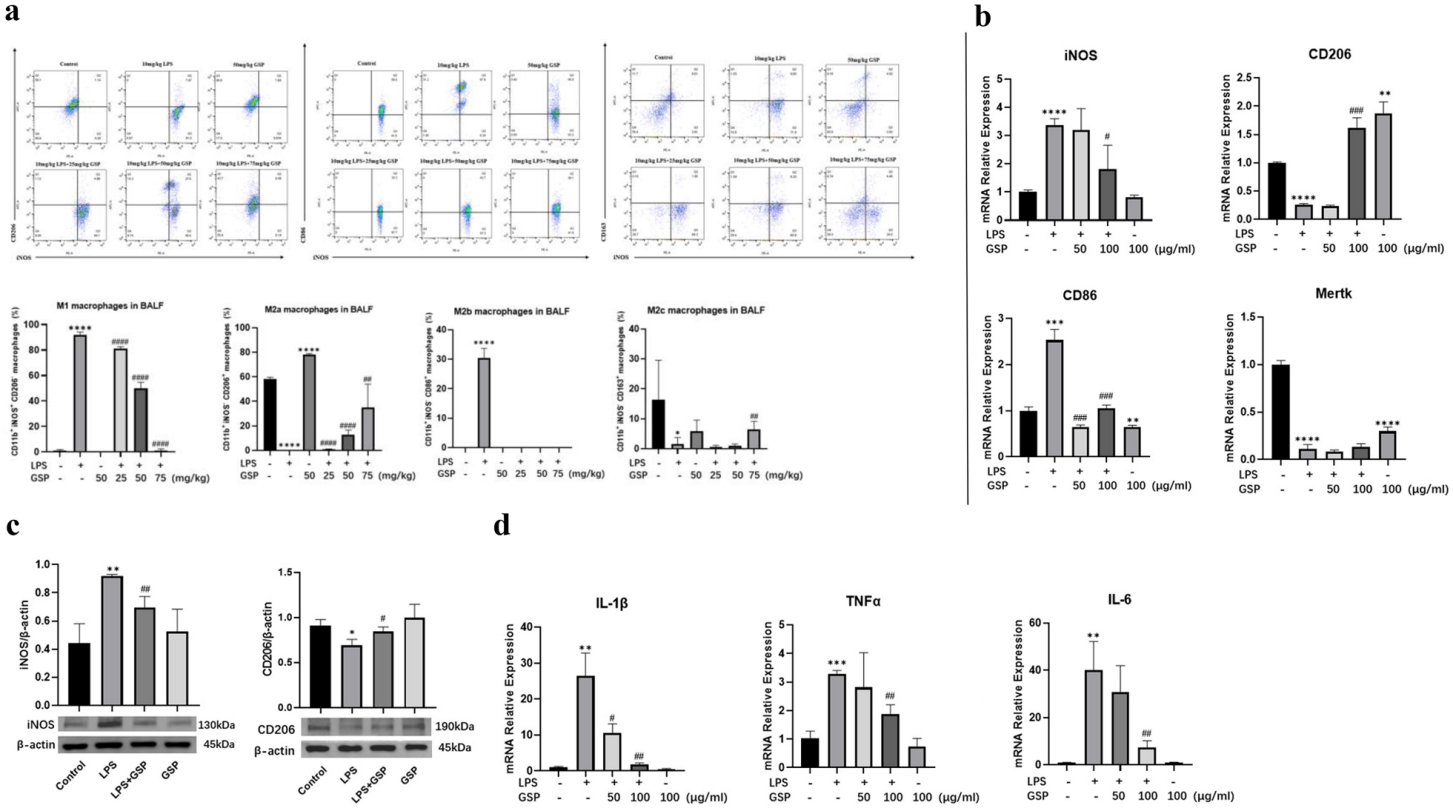
The effect of GSP on macrophage subpopulations and polarity after LPS administration. **a** Flow cytometry analysis of the percentage of CD11b^+^iNOS^+^CD206^−^M1 macrophages, CD11b^+^iNOS^−^CD206^+^M2a macrophages, CD11b^+^iNOS^−^CD86^+^M2b macrophages, and CD11b^+^iNOS^−^CD163^+^M2c macrophages in BALF. **b** mRNA expression of M1 marker (iNOS), M2a/b/c marker genes (CD206/CD86/Mertk). Cells were pretreated with 50 or 100 μg/ml GSP for 24 h and then stimulated with 1 μg/mL LPS for another 3 h. **c** protein expression of iNOS and CD206. Cells were pretreated with 100 μg/ml GSP for 24 h and then stimulated with 1 μg/mL LPS for another 3 h. **d** mRNA expression of IL-1β, TNF-α, and IL-6 genes. Cells were pretreated with 50 or 100 μg/ml GSP for 24 h and then stimulated with 1 μg/mL LPS for another 3 h. The results were the means ± SD. ^*^p < 0.05, ^**^p < 0.01, ^***^p < 0.001, ^****^p < 0.0001 vs. the Control group; ^#^*p* < 0.05, ^##^*p* < 0.01, ^###^*p* < 0.001, ^####^*p* < 0.0001 vs. the LPS group.

We also investigated the percentage of different polarized macrophages in BALF by flow cytometry. As shown in Fig. 3A, the percentage of CD11b^+^iNOS^+^CD206^−^ M1 and CD11b^+^iNOS^−^CD86^+^ M2b lung macrophages was significantly increased, while the percentage of CD11b^+^iNOS^−^CD206^+^ M2a and CD11b^+^ iNOS^−^CD163^+^ M2c lung macrophages was decreased after LPS administration. GSP injection at 25, 50, and 75 mg/kg inhibited M1 macrophage polarization and promoted M2a macrophage polarization; however, only GSP at 75 mg/kg promoted M2c macrophage polarization in the lungs of mice subjected to LPS.

In addition, GSP also suppressed M1 macrophage activation while promoting M2a phenotype macrophage polarization in LPS-induced MH-S cells. GSP did not cause cytotoxicity in MH-S cells at concentrations up to 100 μg/ml (Fig. S4). GSP (100 μg/ml) prevented the LPS-induced M1 phenotype marker iNOS mRNA expression (Fig. 3B). Conversely, GSP (100 μg/ml) significantly reversed the LPS-induced downregulation of the M2a marker (CD206) rather than the M2b (CD86) and M2c (Mertk) marker mRNA expressions (Fig. 3B). Similarly, GSP (100 μg/ml) suppressed LPS-induced iNOS protein expression or enhanced CD206 protein expression in MH-S cells (Fig. 3C). In addition, GSP (100 μg/ml) suppressed LPS-induced IL-1β, TNF-α, and IL-6 mRNA expression increasing in MH-S cells (Fig. 3D). These data indicated that GSP predominately promoted macrophage shift M1 to M2a subtype *in vivo* and *in vitro*.

### Elevating TREM2 By GSP Promoted Macrophage Shift M1 to M2a Phenotype and Inhibited Inflammation

Our data provided compelling evidence of a protective effect of GSP by modulating macrophage transition from M1 to M2a in the experimental LPS-dependent ALI model. However, molecular mechanisms triggered by GSP remain unknown. Next, we used bioinformatics analysis to predict potential targets, which provided directions for further molecular mechanism studies.

#### Identification of DEGs

The principal component analysis (PCA) results showed that the repeatability of data in GSE61298 is fine (Fig. S5A). DEGs of the M2a- and M1-polarized macrophages in the GSE61298 expression profiling datasets were analyzed by using the GEO2R tool. In this dataset, 3207 differentially expressed genes were screened, including 1734 upregulated and 1473 downregulated genes. The adjusted *P* value in the processed data was converted to -log10. These results were drawn from a volcano map (Fig. S5B).

#### Identification of TREM2 As a Candidate Target for Regulating M2a Macrophage Polarization

By applying the cutoffs of relevance score > 10, 1454 ALI-associated candidate targets in the GeneCards dataset were selected. We selected the top 600 DEGs in GSE 61298 for subsequent analysis to investigate the molecular mechanism of macrophage polarization in ALI. A Venn diagram showed that 109 out of 1454 candidate targets responding to ALI treatment were DEGs for M2a/M1 macrophage polarization (Fig. S5C), of which 84 were down-regulated genes and 25 were up-regulated in M2a macrophages (Fig. S5D). As shown in Fig. S5E, GO enrichment analysis revealed that the most significant GO enriched terms related to BPs, such as response to oxygen-containing compound, response to an organic substance, inflammatory response and involvement in the cell surface, external side of the plasma membrane, and extracellular region, as well as mainly receptor binding, cytokine receptor binding, cytokine activity, and other molecular roles.

In addition, among the 109 intersection genes, 35 were identified to associate with a response to lipopolysaccharide, 49 were identified to a response to stress, and 20 were related to acute inflammatory response, implying critical roles in the pathogenesis of ALI (Table S1). Then we took the intersection of these genes involved in the above biological process and obtained 8 candidate targets (Fig. S6). The detailed parameters were shown in Table S2. We found that only TREM2 expression was upregulated in M2a macrophages. We hypothesized that TREM2-associated M2a polarization played a critical role in the inflammation process in ALI.

#### GSP Promoted TREM2 Expression in Lung Macrophages of ALI Mice

**Fig. 4:**
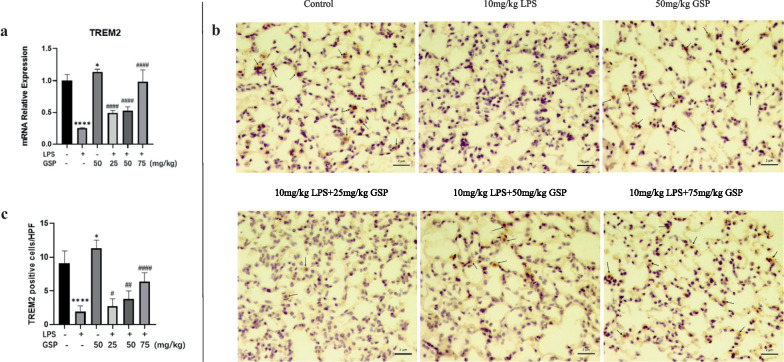
GSP promoted TREM2 expression in lung macrophages of ALI mice. **a** GSP promoted TREM2 gene expression in primary mouse alveolar macrophages under the LPS challenge. **b**-**c** Immunohistochemistry of the TREM2 protein in lung macrophages (magnification 400 × , as indicated by the black arrows). n = 5/group. The results are the means ± SD. ^*^p < 0.05, ^****^p < 0.0001 vs. the Control group; ^#^*p* < 0.05, ^##^*p* < 0.01, ^####^*p* < 0.0001 vs. the LPS group.

According to the results of bioinformatics analysis, we predicted that TREM2 was involved in LPS-induced ALI. Hence, the expression of TREM2 in ALI mice was measured by RT-PCR and IHC. As expected, the LPS-treated group showed a significant decrease in the levels of TREM2 expression in primary mouse lung macrophages compared with the control group, while GSP (25, 50, and 75 mg/kg) pretreatment led to a significant increase in TREM2 mRNA expression (Fig. 4A). We also found the number of TREM2-positive cells was notably decreased in the LPS-induced ALI group compared to the control group, and GSP (25, 50, and 75 mg/kg) increased the number of TREM2-positive cells, indicating that GSP treatment promoted TREM2 expression in lung macrophages of ALI mice (Fig. 4B, C).

#### TREM2 was Involved in GSP-mediated M2a Macrophage Polarization and Anti-inflammatory Activity in MH-S Cells

**Fig. 5:**
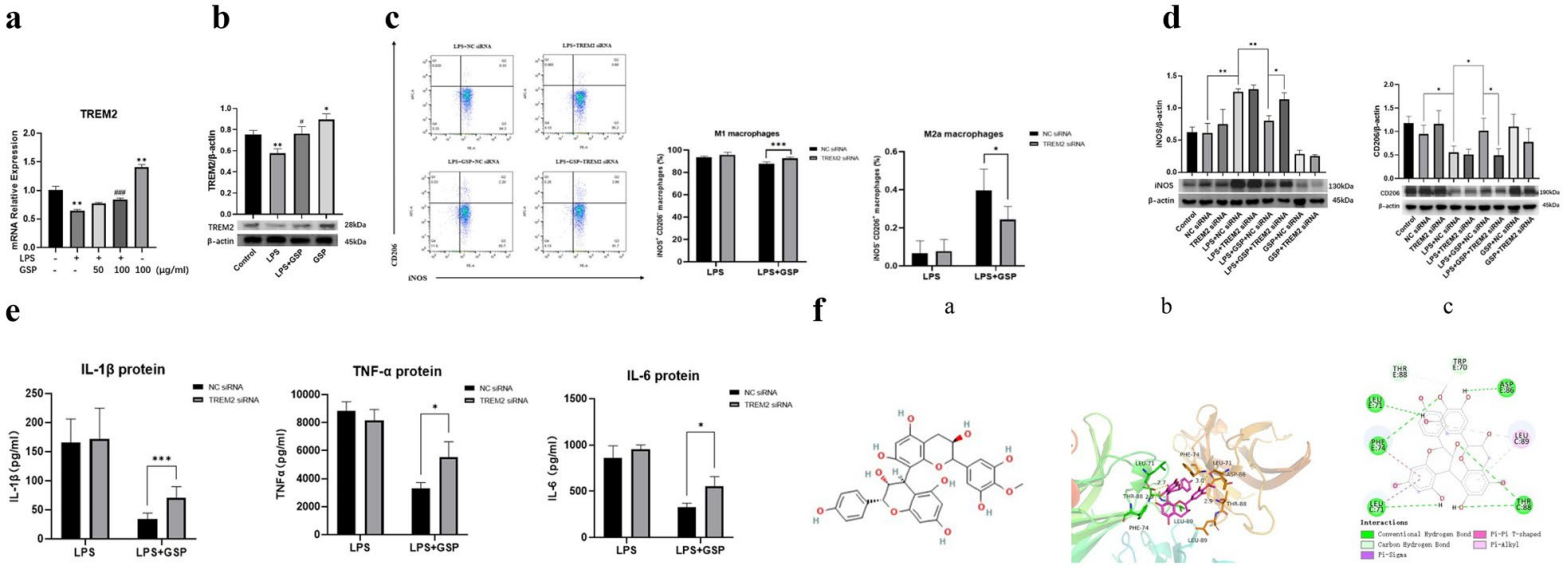
TREM2 was involved in GSP-mediated M2a macrophage polarization and anti-inflammatory activity in MH-S cells. **a**-**b** The expression of TREM2 at the gene transcription and protein levels. Cells were pretreated with 50 or 100 μg/ml GSP for 24 h and then stimulated with 1ug/mL LPS for another 3 h. The results were the means ± SD. ^*^p < 0.05, ^**^p < 0.01 vs. the Control group; ^#^*p* < 0.05, ^###^*p* < 0.001 vs. the LPS group. **c** Flow cytometry analysis of the percentage of iNOS^+^CD206^−^ M1 macrophages and iNOS^−^CD206^+^ M2a macrophages. 24 h after siRNA transfection, cells were pretreated with 100 μg/ml GSP for 24 h and then stimulated with 1 μg/mL LPS for another 3 h. **d** The expression of M1 marker iNOS and M2a marker CD206 at the protein levels. 24 h after siRNA transfection, cells were pretreated with 100 μg/ml GSP for 24 h and then stimulated with 1 μg/mL LPS for another 3 h. **e** Pro-inflammatory cytokine IL-1β, TNF-α, and IL-6 protein levels in the cell culture supernatant. 24 h after siRNA transfection, cells were pretreated with 100 μg/ml GSP for 24 h and then stimulated with 1ug/mL LPS for another 3 h. **f** Molecule docking. **a** Chemical structure of GSP. **b** 3D molecular docking model of the GSP with TREM2. **c** 2D virtual molecular docking of the GSP with TREM2. The results were the means ± SD. ^*^p < 0.05; ^**^p < 0.01; ^***^p < 0.001.

RT-PCR and western blot consistently showed that the TREM2 gene and protein expressions were decreased in the LPS-stimulated MH-S cells compared with the Control group (Fig. 5A, B). GSP (100 μg/ml) pretreatment markedly enhanced the gene and protein expression of TREM2 and prevented the LPS-induced decrease in the TREM2 gene and protein expression in MH-S cells (Fig. 5A, B). To confirm that TREM2 was involved in the GSP-mediated macrophage polarization and inflammation in LPS-induced MH-S cells, we used siRNA for TREM2 knockdown. TREM2 protein expression was significantly decreased in MH-S cells transfected with TREM2 siRNA for 24 h (Fig. S7). Upon knockdown of TREM2, the mRNA expression levels of M1 marker iNOS increased significantly in the GSP (100 μg/ml) treatment group, while the mRNA expression levels of M2a marker CD206 decreased significantly (Fig. S8). Then, we performed further flow cytometry and western blotting tests. We found that TREM2 knockdown by siRNA significantly increased the percentage of M1 macrophages in the GSP (100 μg/ml) treatment group, while significantly decreasing the percentage of M2a macrophages (Fig. 5C). Similarly, as shown in Fig. 5D, TREM2 knockdown by siRNA significantly increased iNOS protein levels and decreased CD206 protein levels in the GSP (100 μg/ml) treatment group. In addition, TREM2 knockdown by siRNA significantly increased the secretion of IL-1β, IL-6, and TNF-α at the protein levels in the GSP (100 μg/ml) treatment group (Fig. 5E).

Finally, we predicted the binding mode of the TREM2 protein and the small molecule compound GSP using molecular docking techniques. Molecular docking scores are based on binding affinity, and when the docking score is smaller, the binding affinity to the target protein is stronger. The docking score <  − 7 kcal/mol indicated a high binding activity between the receptor and ligand [29]. We found that the TREM2-binding domain was identified in amino acids 71,74,86,88,89 of GSP, and the docking score was 9.47 kcal/mol. As shown in Fig. 5F, the docking models indicate a strong binding effect between GSP and TREM2. Taken together, TREM2 was a potential target for GSP to perform its biological functions, including alleviating LPS-induced inflammatory response and promoting M2a polarization.

### TREM2 Regulated the Activation of the PI3K/Akt Signaling Pathway in the Effects of GSP in MH-S Cells

**Fig. 6:**
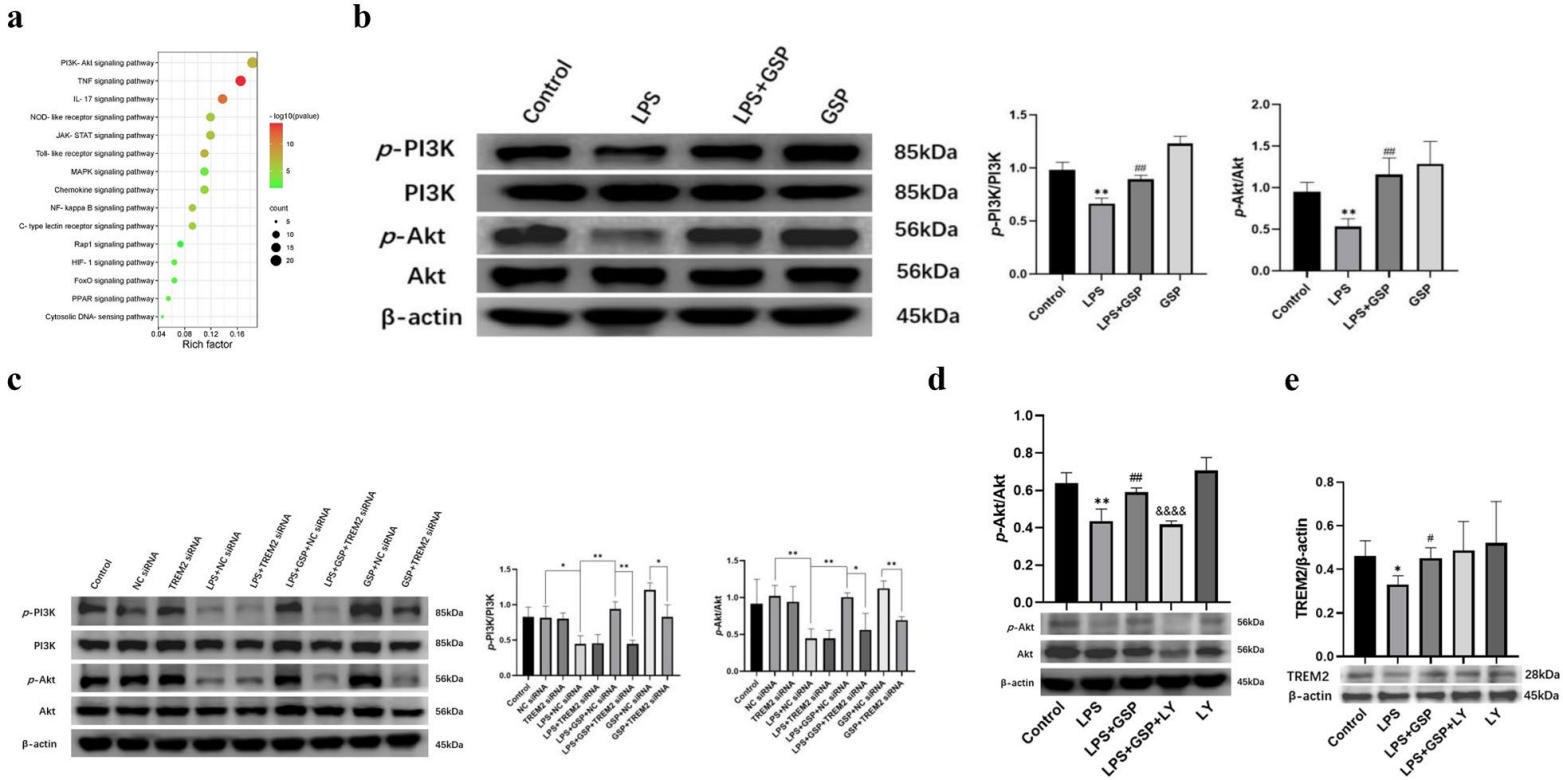
TREM2 regulated the activation of the PI3K/Akt signaling pathway in MH-S cells. **a** Kyoto Encyclopedia of Genes (KEGG) pathway enrichment analysis. **b** The PI3K/Akt phosphorylation protein expression. Cells were pretreated with 100 μg/ml GSP for 24 h, and then stimulated with 1 μg/mL LPS for another 3 h. **c** The PI3K/Akt phosphorylation protein expression after siRNA transfection (^*^p < 0.05, ^**^p < 0.01). Cells transfected with 50 nM TREM2 siRNA for 24 h were treated with 100 μg/ml GSP for 24 h and then exposed to 1 μg/ml LPS for another 3 h. **d** Effects of PI3K inhibitor LY294002(LY) on *p*-Akt/Akt expression. Cells were pretreated with 100 μg/ml GSP for 22 h, treated with 20 μM LY294002 for 2 h, and then exposed to LPS for another 3 h. **e** Effects of LY294002 on TREM2 expression. Cells were pretreated with 100 μg/ml GSP for 22 h, treated with 20 μM LY294002 for 2 h, and then exposed to LPS for another 3 h. The results were the means ± SD. ^*^p < 0.05, ^**^p < 0.01 vs. the control group; ^#^*p* < 0.05, ^##^*p* < 0.01 vs. the LPS group. ^&&&&^*p* < 0.0001 vs. the LPS + GSP group.

As mentioned above, KEGG enrichment analysis of 109 DEGs involved in ALI showed that they were strongly correlated with the “PI3K/Akt signaling pathway”, “TNF signaling pathway”, and “IL-17 signaling pathway” (Fig. 6A). Studies had shown that these signaling pathways were associated with hypoxia, inflammation, and autophagy [30–32], providing a reliable direction for molecular mechanisms. Most importantly, the number of intersection genes related to the PI3K/Akt signaling pathway was the largest. It hasd been reported that the PI3K/Akt signaling pathway was a co-upstream of the TNF and IL-17 signaling pathways or might be closely related to them [33, 34]. Our results showed that the expression of *p*-PI3K and *p*-Akt protein, two major markers of the activated PI3K/Akt signaling pathway, were both decreased in LPS-treated MH-S cells, and GSP (100 μg/ml) pretreatment markedly enhanced the protein expression of *p*-PI3K and *p*-Akt (Fig. 6B), suggesting that GSP could activate the PI3K/Akt signaling pathway. TREM2 knockdown by siRNA blocked the GSP-enhanced PI3K and Akt protein phosphorylation (Fig. 6C). In contrast, the PI3K inhibitor LY294002 blocked the GSP-enhanced *p*-Akt protein (Fig. 6D) but had no promoting or inhibiting effect on TREM2 expression in MH-S cells (Fig. 6E), indicating that TREM2 acted as an upstream mediator of the PI3K/Akt signaling pathway in the effects of GSP in MH-S cells.

### GSP Protected Against LPS-induced ALI in Mice By Activating the PI3K/Akt Signaling Pathway

**Fig. 7:**
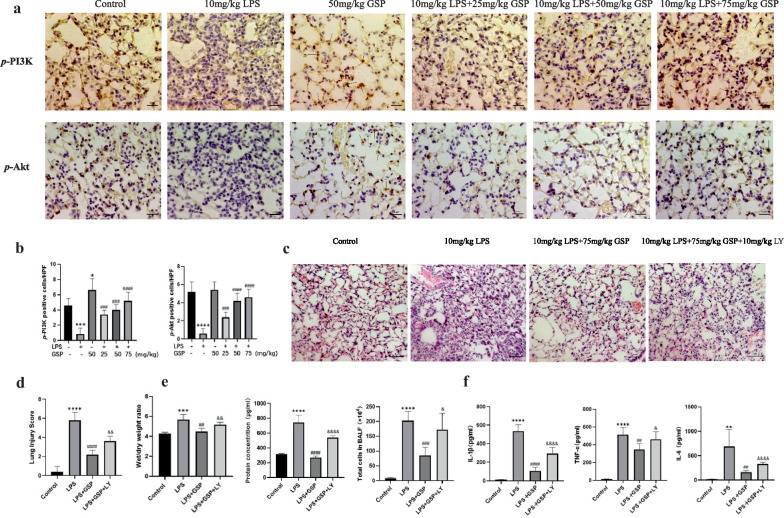
GSP protected against LPS-induced ALI in mice by activating the PI3K/Akt signaling pathway. **a** Immunohistochemistry staining of *p*-PI3K and *p*-Akt proteins in lung macrophages (magnification 400 × , as indicated by the black arrows). **b** Immunohistochemical analysis of *p*-PI3K and *p*-Akt positive macrophages. **c** Effects of PI3K inhibitor LY294002 (LY) on pathological changes of lung tissue. Sections of lung tissues were stained with H&E (magnification 200 ×). **d** Effects of PI3K inhibitor LY294002 on lung injury score. **e** Effects of PI3K inhibitor LY294002 on lung wet/dry (W/D) weight ratio, the total protein concentration in the supernatant, and the total cell count in BALF. **f** Effects of PI3K inhibitor LY294002 on IL-1β, TNF-α, and IL-6 levels in BALF. n = 5. The values represented the means ± SD. ^*^p < 0.05, ^**^p < 0.01, ^***^p < 0.001, ^****^p < 0.0001 vs. the control group; ^##^*p* < 0.01, ^###^*p* < 0.001, ^####^*p* < 0.0001 vs. the LPS group. ^&^*p* < 0.05, ^&&^*p* < 0.01, ^&&&&^*p* < 0.0001 vs. the LPS + GSP group.

Immunohistochemical staining showed that the *p*-PI3K and* p*-Akt-positive macrophages in ALI samples were significantly lower than in the healthy control. Conversely, compared with the model group, *p*-PI3K and *p*-Akt positive macrophages were significantly increased in the GSP (25, 50, and 75 mg/kg) treatment group (Fig. 7A, B). These results suggested that the PI3K/Akt signaling pathway was inhibited in ALI and that GSP could activate the PI3K/Akt signaling pathway. Moreover, to confirm whether the protective effects of GSP at a dose of 75 mg/kg against ALI were attenuated when the PI3K/Akt signaling pathway was inhibited, we tested the role of PI3K inhibitor LY294002 (LY) in pathological changes of lung tissue, lung capillary barrier function, and lung inflammation. As expected, some evaluation indicators in LPS + GSP + LY group, including pathological changes, lung injury score, W/D weight ratio, pro-inflammatory factors levels in BALF, total cell counts in BALF, and protein concentration in BALF, were significantly increased compared with the LPS + GSP group (Fig. 7C–F), suggesting that LY294002 abolished the protective effect of GSP on ALI. Furthermore, immunohistochemical staining showed that LY294002 inhibited the GSP-enhanced *p*-Akt expression (Fig. S9). Taken together, LY294002 could block the PI3K/Akt signaling pathway and interfered with the anti-ALI effect of GSP.

### GSP-mediated PI3K/Akt signaling activation regulated macrophage M1/M2a Polarization *In Vivo* and *In Vitro*

**Fig. 8:**
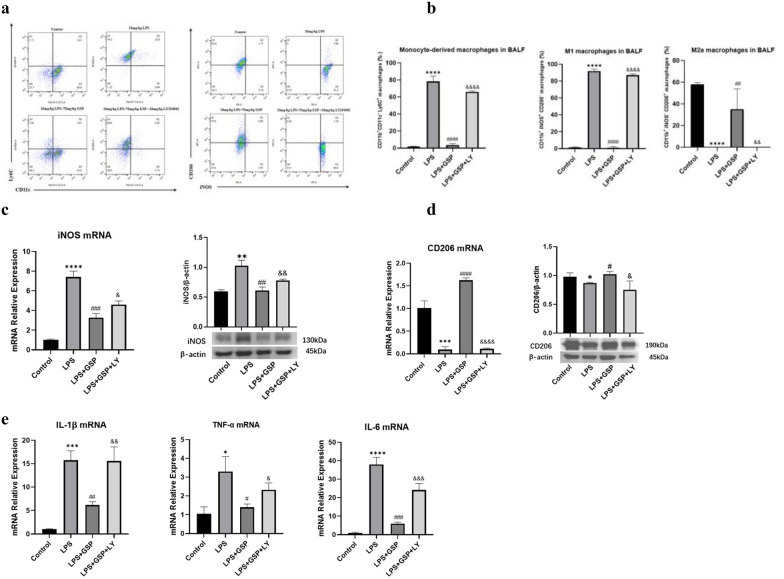
Effects of PI3K inhibitor LY294002 on M2a/M1 macrophage polarization and inflammation regulated by GSP *in vivo* and *in vitro*. **a**-**b** Flow cytometry analysis of the percentage of CD11b^+^CD11c^−^Ly6C^+^ monocyte-derived macrophages, CD11b + iNOS + CD206- M1 lung macrophages and CD11b + iNOS-CD206 + M2a lung macrophages in BALF of mice. **c** The effect of LY294002 on M1 marker iNOS mRNA and protein expression of MH-S cells. **d** The effect of LY294002 on M2a marker CD206 mRNA and protein expression of MH-S cells. **e** The effect of LY294002 on IL-1β, IL-6, and TNF-α mRNA levels of MH-S cells. Cells pretreated with 100 μg/ml GSP for 22 h were treated with 20 μM LY294002 for 2 h and then exposed to LPS for another 3 h. The results were the means ± SD. ^*^p < 0.05, ^**^p < 0.01, ^***^p < 0.001, ^****^p < 0.0001 vs. the Control group; ^#^*p* < 0.05, ^##^*p* < 0.01, ^###^*p* < 0.001, ^####^*p* < 0.0001 vs. the LPS group; ^&^*p* < 0.05, ^&&^*p* < 0.01, ^&&&^*p* < 0.001, ^&&&&^*p* < 0.0001 vs. the LPS + GSP group.

To further confirm that the PI3K/Akt pathway was involved in the effect of GSP on the mobilization of inflammation and the regulation of M1/M2a macrophage polarization, we used LY294002 to block the PI3K/Akt pathway *in vivo* and *in vitro* experiment. We found that PI3K inhibition by LY294002 reversed the GSP-mediated decrease in the percentage of monocyte-derived macrophages and M1 macrophages, and the increase in M2a macrophages in BALF of mice (Fig. 8A, B). In addition, LY294002 also blocked the GSP-mediated decrease in M1 marker iNOS mRNA and protein expression (Fig. 8C) and the increase in M2a marker CD206 gene and protein expression in MH-S cells (Fig. 8D). Besides, as shown in Fig. 8E, the downregulated IL-1β, TNF-α, and IL-6 mRNA levels in GSP-pretreated MH-S cells were reversed when given LY294002.

## Discussion

**Fig. 9:**
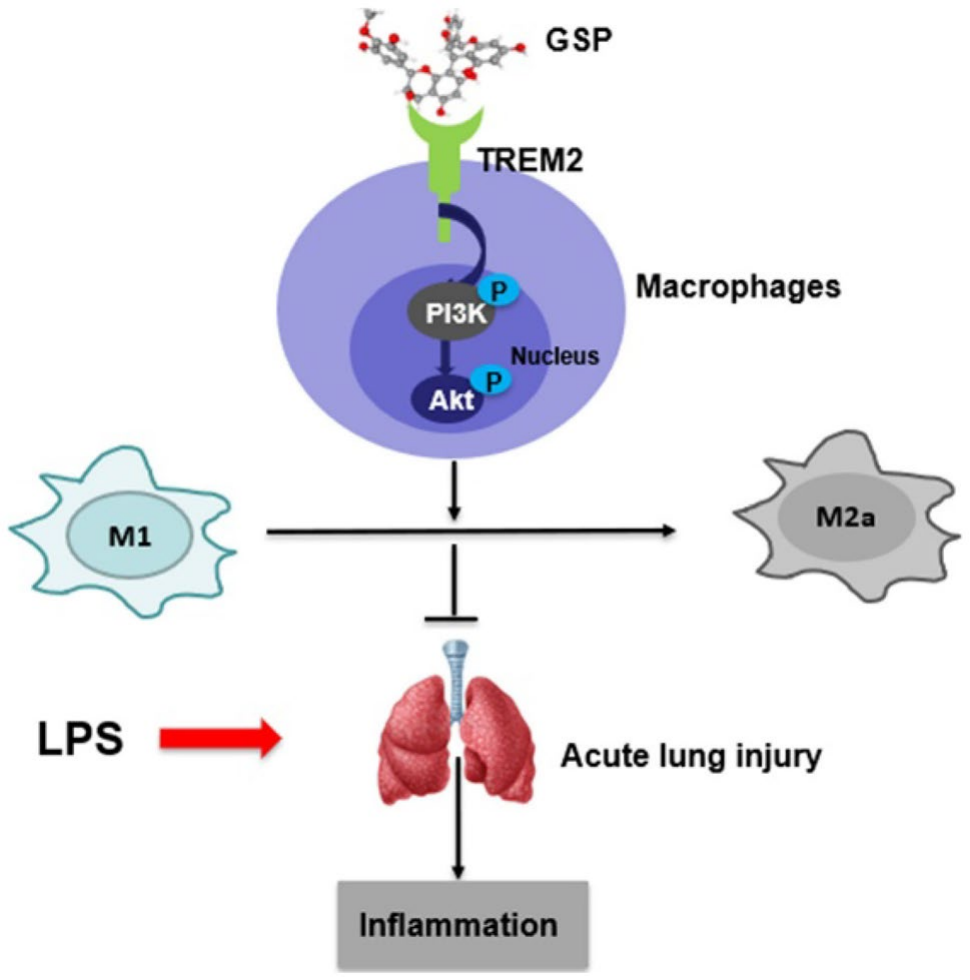
The proposed mechanism of GSP in modulating macrophage polarization in the LPS-induced ALI mouse model. GSP combined to TREM2 and promoted the phosphorylation of PI3K/Akt pathway to modulate M2a macrophage polarization and alleviated LPS-induced ALI in mice.

The current study was designed to test the hypothesis that GSP significantly promoted alternative M2 activation of macrophages to ameliorate LPS-induced ALI and clarify this effect's molecular mechanisms. A proposed mechanism is depicted in Fig. 9. Our results demonstrated that GSP protected against LPS-induced ALI in mice and predominately stimulated M2a alternative polarization of lung macrophages *in vivo* and *in vitro*. In addition, based on bioinformatics analysis, molecular docking, and experimental verification, this study identified TREM2 as a potential target of GSP, regulating the PI3K/Akt pathway signaling activation, which played a crucial role in GSP-mediated M2a macrophage polarization and anti-inflammatory effects in a mouse model of LPS-induced ALI.

Macrophage polarization is critical in ALI pathogenesis. It can be seen in experimental ALI, which involves an extended or magnified M1 response and faulty M2-mediated repair [12, 13]. Therefore, targeting macrophage polarization to treat ALI is of interest. GSP, a polyphenol compound isolated from grape seeds, has many biological activities such as anti-apoptotic, antioxidant, and anti-inflammatory, and exhibits a variety of therapeutic capacities in obesity, cancer, diabetes complications, and cardiac dysfunction after myocardial infarction [16–22]. However, the role of GSP in macrophage polarization-mediated acute pulmonary inflammation has not been fully characterized. In our study, we proved that GSP protects against the LPS-induced ALI mice model by exerting several synergistic actions, including inhibition of interstitial edema, maintenance of the alveolar-capillary barrier, and reduction of cytokine production, which might be achieved by altering the macrophage phenotype. On the one hand, GSP alleviated the recruitment of monocyte-derived macrophages to the lung and reduced the mobilization of pulmonary inflammatory response. On the other hand, GSP at different doses (25, 50, and 75 mg/kg) significantly promoted M2a polarization, while only 75 mg/kg GSP promoted M2c polarization of murine lung macrophages. Consistently, GSP promoted M2a but not M2b or M2c macrophage polarization in LPS-induced MH-S cells. Therefore, the promotion of M2a polarization by GSP was more effective than M2c in protecting against pulmonary inflammation and lung injury.

The possible targets regulated by GSP were predicated and confirmed using bioinformatics analysis, molecular docking technology, and *in vitro* and *in vivo* experiments. We identified that TREM2 was a common target between M2a/M1 macrophage polarization and ALI. TREM2, a major family member of the super immunoglobulin family known as TREM, selectively expressed on macrophages. TREM2 has been extensively studied in microglial cells that exert anti-inflammatory properties and promotes phagocytosis of apoptotic neuronal cells in neurodegenerative diseases [35–38]. A previous study revealed that TREM2 activation promoted microglial switching from the detrimental M1 phenotype to the beneficial M2 phenotype and that, as a result, decreased the number of apoptotic neurons after ischemic damage [39]. In our study, TREM2 expression was downregulated in LPS-induced M1 polarized primary mouse lung macrophages and MH-S cells, while GSP pretreatment upregulated the expression of TREM2 accompanied by upregulated expression of M2a marker. The TREM2 knockdown decreased the effects of GSP on M2a macrophage polarization and inflammation elimination. These findings suggested that TREM2 was involved in GSP-mediated M2a macrophage polarization in alleviating ALI.

In the KEGG enrichment assay, we observed that PI3K/Akt signaling pathway was a critical pathway in regulating M1/M2a macrophage polarization for ALI treatment, indicating a potential to be a target. Subsequently, we verified whether the biological action of GSP in ALI and macrophage was associated with activating the PI3K/Akt signaling pathway. *In vivo* and *in vitro* models, GSP robustly increased the activity of the PI3K/Akt signaling pathway. Inhibition of the PI3K/Akt signaling pathway weakened the protective effects of GSP in ALI mice as well as blocked the GSP-induced M2a polarization of macrophages in primary lung macrophages of ALI mice and LPS-stimulated MH-S cells, suggesting that the promotion of M2a polarization by GSP to exert anti-inflammatory effects might be mediated by the activated PI3K/Akt signaling pathway.

Studies had reported that TREM2 could associate with the adaptor DAP12, mediating downstream signaling through a cytoplasmic ITAM domain, which could recruit Syk and activated PI3K, phospholipase C, and Vav signaling cascades, and that the PI3K/Akt signaling was the downstream target of TREM2 and was involved in TREM2-mediated neuroinflammatory response [40, 41]. To explore the relationship between TREM2 and the PI3K/Akt signaling pathway in GSP-induced M2a polarization of macrophages, we knocked down TREM2 with siRNA. It could completely block GSP-induced *p*-PI3K and *p*-Akt expression in LPS-stimulated MH-S cells. In contrast, the PI3K inhibitor LY294002 had no promotive or suppressive effect on TREM2 expression, suggesting that TREM2 acted as an upstream mediator of the PI3K/Akt signaling pathway in the effects of GSP in MH-S cells.

Notably, the beneficial effects of GSP in the ALI of mice subjected to LPS were following with its regulation of macrophage polarization, suggesting that the promotion of macrophage polarization to the M2a anti-inflammatory phenotype might be responsible for the beneficial effects of GSP in our *in vivo* study. However, our results in the *in vivo* study did not exclude the possibility that GSP may affect other cells, such as T and B cells. There were also several limitations in this study. First, the KEGG enrichment assay showed that multiple signaling pathways such as “TNF signaling pathway” and “IL-17 signaling pathway” were also crucial other than the “PI3K/Akt pathway” and whether GSP could alleviate ALI through other signal transduction pathways had not been fully elucidated in this study. Second, the isolated BALF contained many types of cells, especially abundant neutrophils in mouse subject to LPS. As a result, the TREM2 gene expression levels measured by RT-PCR didn't entirely derive from lung macrophages. Similarly, due to the lack of macrophage markers in IHC, the indicated *p*-PI3K- and *p*-Akt- positive cells could not fully represent macrophages. Third, GSP increased TREM2 expression and bonded with TREM2 strongly. Further verification that GSP is a ligand for TREM2 should be performed by measuring the activity of the TREM2-GSP binding site in the future. Finally, we only used LY294002 and siRNA to inhibit PI3K/Akt pathway and TREM2, respectively, to verify their relationship. Adding agonists or overexpressing TREM2 and transgenic animal studies should be conducted in the future for better mechanics exploration regarding macrophage polarization.

## CONCLUSION

Overall, the current study first demonstrated that GSP played a series of synergistic roles to protect LPS-induced ALI mouse models, including inhibiting pathological damage such as pulmonary interstitial edema, bleeding, and inflammatory cell infiltration, protecting alveolar-capillary barrier function, and reducing the production of inflammatory cytokines. These protective effects of GSP might be attributed to activating the TREM2/PI3K/Akt signaling pathway to promote macrophage polarization from M1 to M2a. Based on these findings, GSP appears to be a promising and attractive treatment option for ALI.

### Supplementary Information

Below is the link to the electronic supplementary material.

Supplementary file1 (DOCX 3362 KB)

## Data Availability

The dataset supporting the conclusions of this article are included within the article.
