# Supplementary material for: Grape Seed Proanthocyanidin Ameliorates LPS-induced Acute Lung Injury By Modulating M2a Macrophage Polarization Via the TREM2/PI3K/Akt Pathway
Source: Inflammation. 2023 Aug 11;46(6):2147–64. doi: 10.1007/s10753-023-01868-5 (PMC10673742; doi:10.1007/s10753-023-01868-5)
Supplement: Supplementary file 1 — Supplementary file1 (DOCX 3362 KB) [file 10753_2023_1868_MOESM1_ESM.docx]

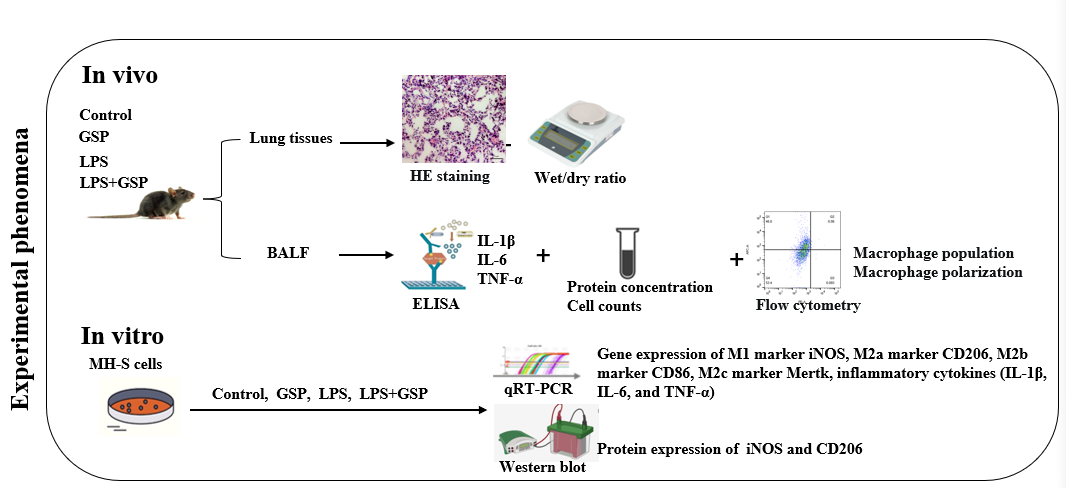


**
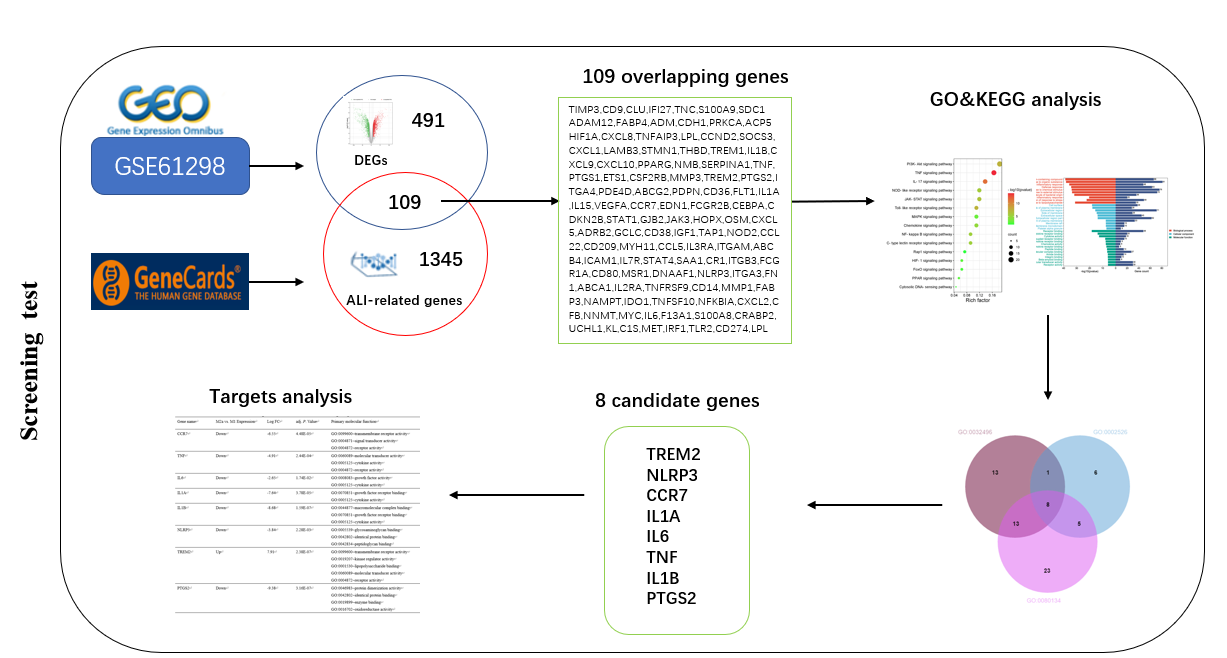
**

**
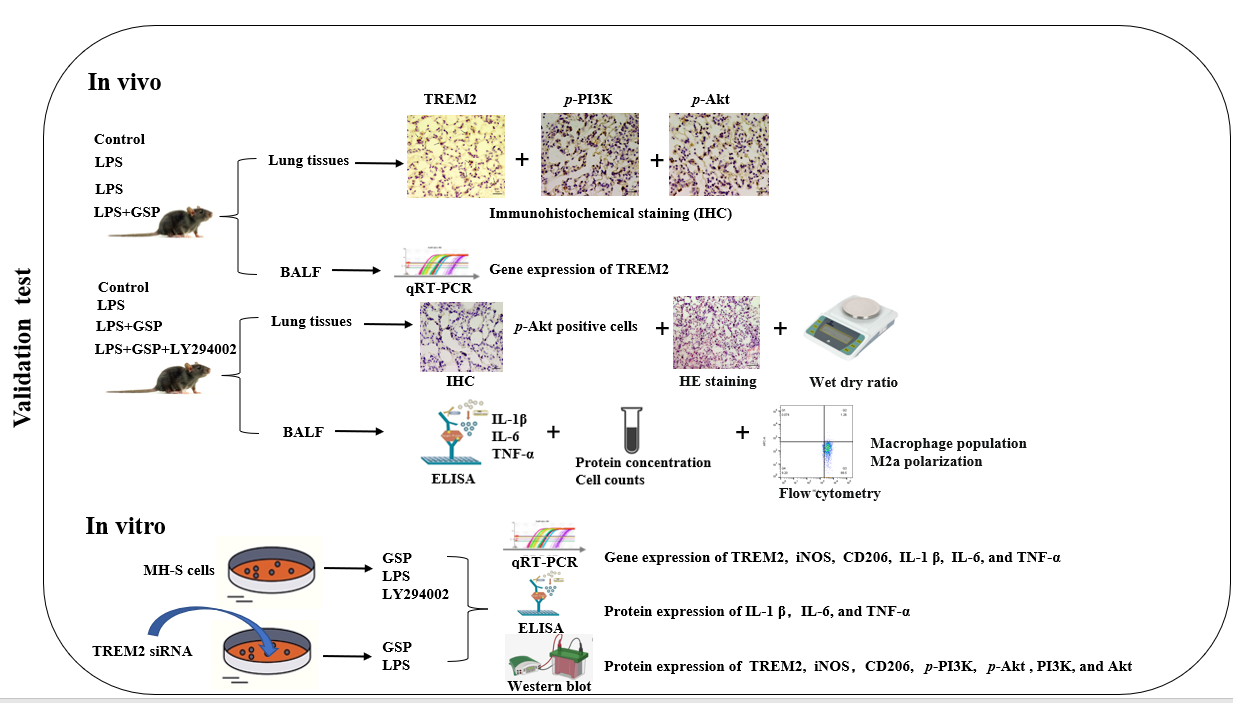
**

**Figure 1. The work-flow of this study.**

**
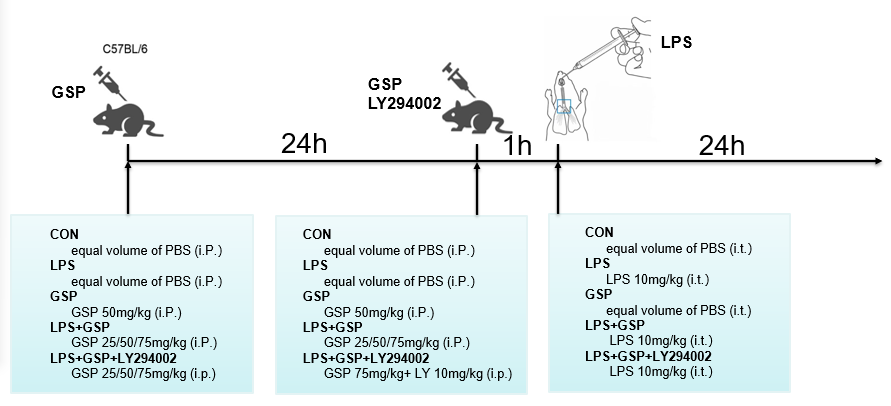
**

**Figure 2. Murine model of LPS‑induced ALI and drug treatment.**

**
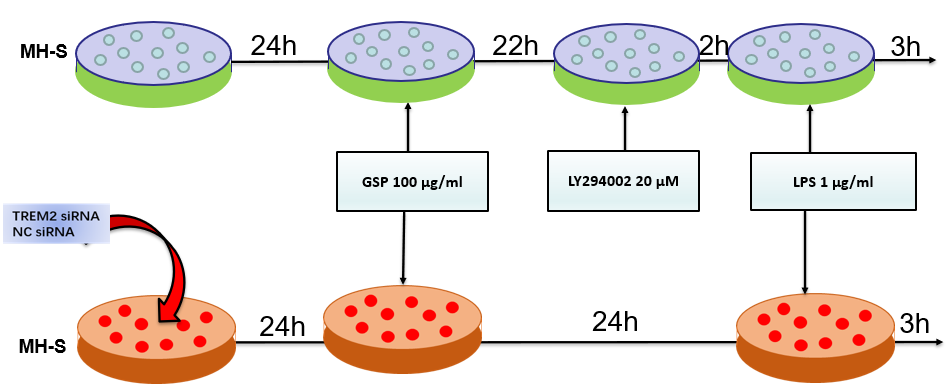
**

**Figure 3. The cell culture and interfering conditions.**

**
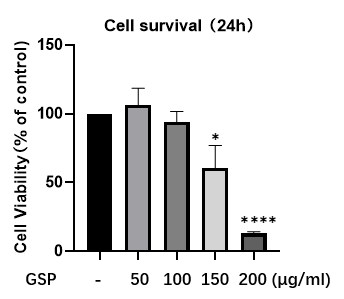
**

**Figure 4. CCK-8 measurement of cell vitality.** Cells were treated with different concentrations of GSP for 24 h.

**C**

**B**


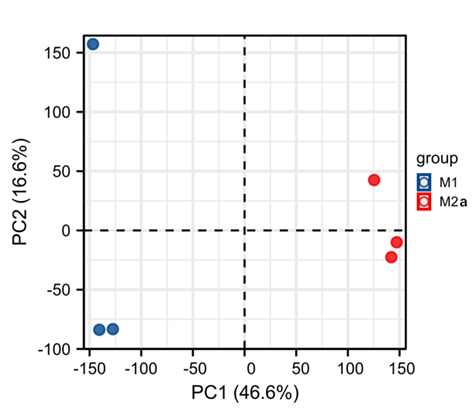

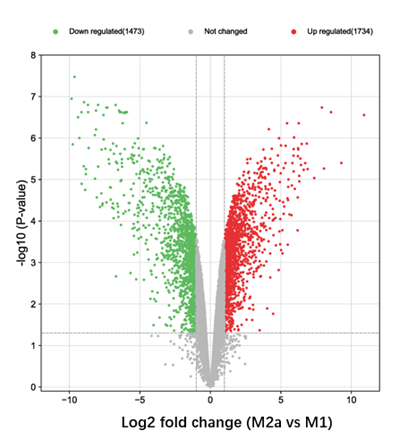

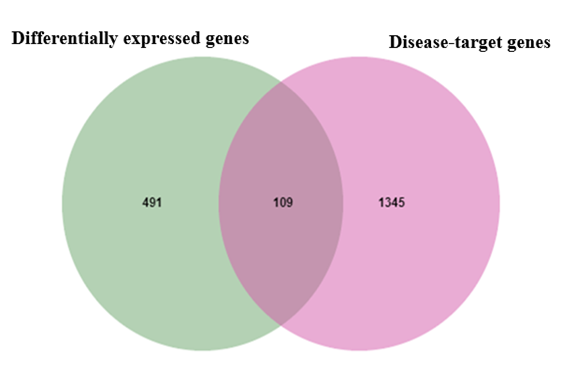

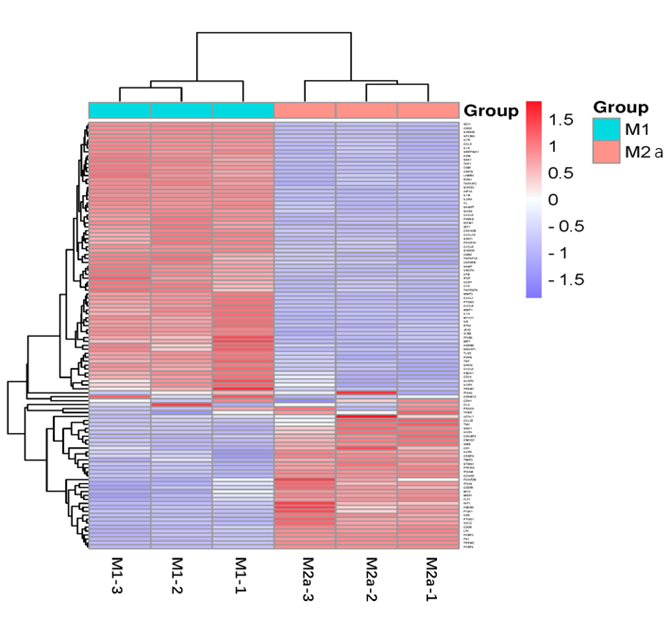

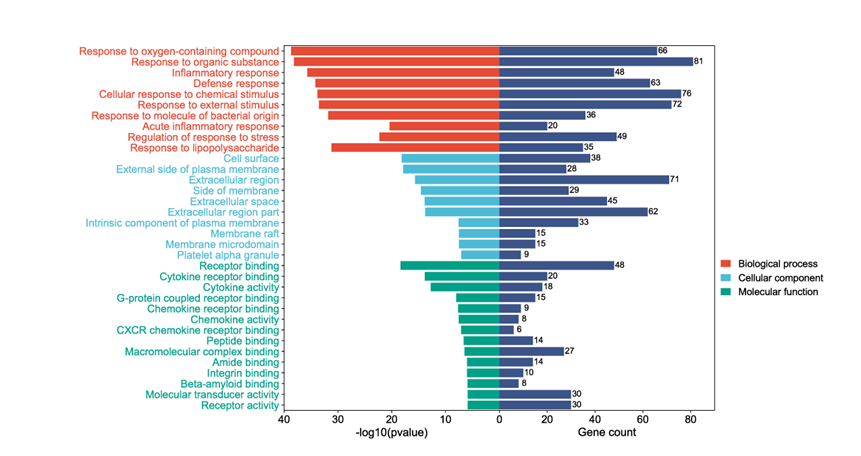


**E**

**D**

**A**

**Figure 5. Differentially expressed genes in M2a- and M1-polarized macrophages.** (A) Principal component analysis for GSE61298. (B) Volcano map of differential genes. Upregulated genes with significant difference are in red, genes that were not significantly different are in grey, and significant downregulated genes are in green. (C) Venn plot of intersection genes. (D) The heat map of the expression of 109 intersection genes. (E) GO enrichment analysis. GO= Gene Ontology.


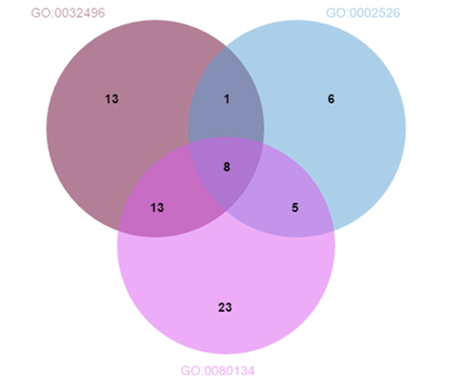


**Figure 6. Venn diagram of intersection among ALI-related biological process** (A) GO:0032496: response to lipopolysaccharide. (B) GO:0080134：regulation of response to stress. (C) GO:0002526: acute inflammatory response.

**
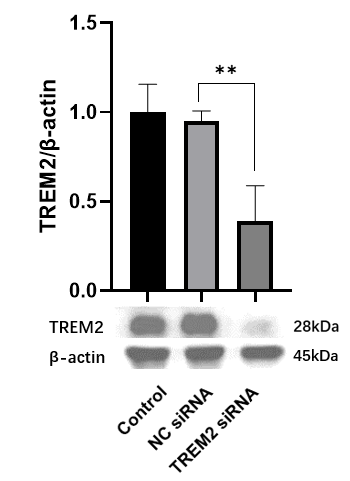
**

**Figure 7. Expression of the TREM2 protein in MH-S cells transfected with 50 nM TREM2 siRNA or negative control (NC) siRNA for 24 h.**

**A B**

**
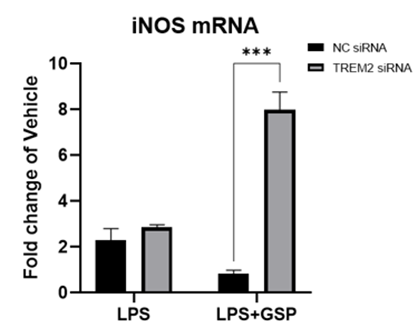

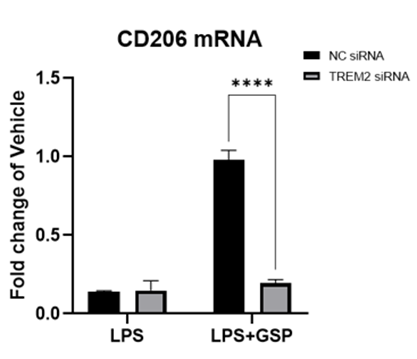
**

**Figure 8.** **The mRNA** **expression levels of (A) M1 marker iNOS and (B) M2a marker CD206 after TREM2 knockdown by a siRNA.** 24h after siRNA transfection, cells were pretreated with 100 μg/ml GSP for 24 h and then stimulated with 1μg/mL LPS for another 3 h. The results were the means ± SD. ^***^*p* < 0.001; ^****^*p*<0.0001.


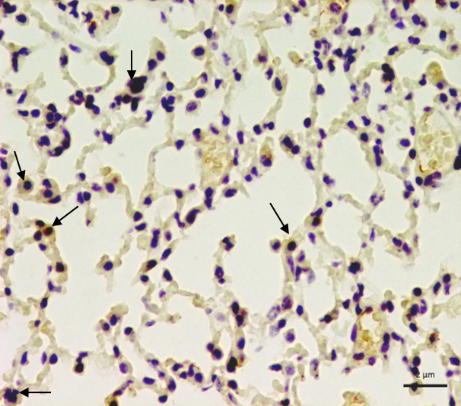


LPS

Control


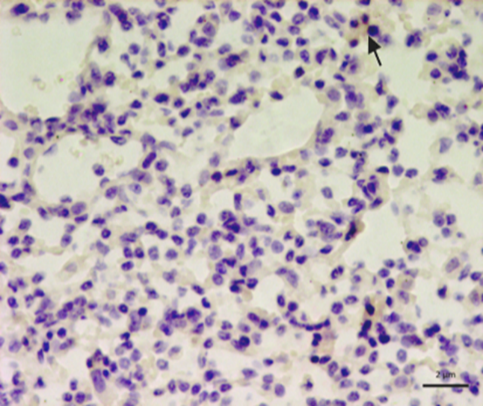


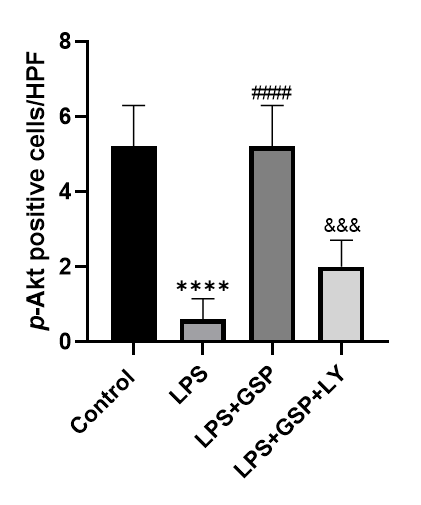


LPS+GSP (75)

LPS+GSP (75) + LY

**
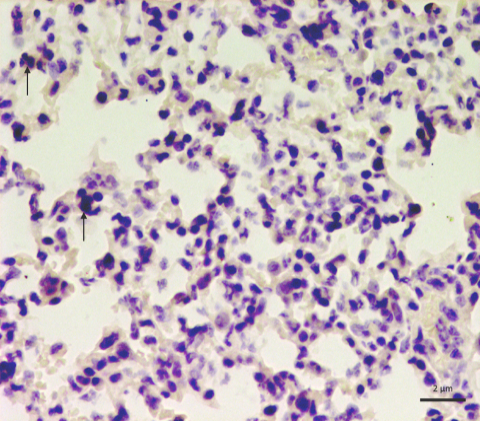

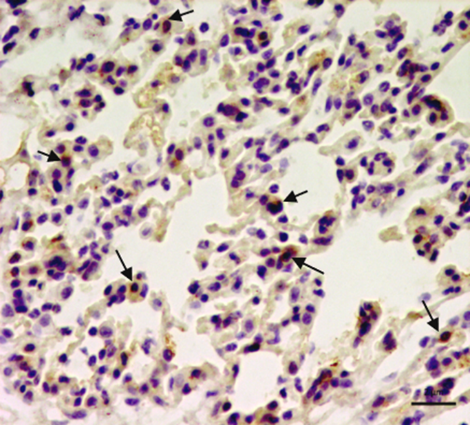
**

**Figure 9. Representative images of *p*-Akt protein level in lung macrophages determined by immunohistochemistry (magnification 400×, as indicated by the black arrows).** n=5.The values represent the means ± SD, ^****^*p* < 0.0001 vs. the Control group; ^####^*p* < 0.0001 vs. the LPS group; ^&&&&^*p* < 0.0001 vs. the LPS+GSP group.

**Table 1. ALI/ARDS‐related GO terms enriched by the 109 intersection genes.**

| **Ontology** | **ID** | **Description** | **Gene ratio** | ***P*‐value** | **Count** |
| --- | --- | --- | --- | --- | --- |
| BP | GO:0032496 | response to lipopolysaccharide | 35/109 | 6.30E-32 | 35 |
| BP | GO:0080134 | regulation of response to stress | 49/109 | 4.99E-23 | 49 |
| BP | GO:0002526 | acute inflammatory response | 20/109 | 4.05E-21 | 20 |

**Table 2. The information on intersection genes involved in ALI-related biological process.**

| Gene name | M2a vs. M1 Expression | Log FC | adj. *P*. Value | Primary molecular function |
| --- | --- | --- | --- | --- |
| CCR7 | Down | -6.55 | 4.48E-05 | GO:0099600~transmembrane receptor activity  GO:0004871~signal transducer activity  GO:0004872~receptor activity |
| TNF | Down | -4.91 | 2.44E-04 | GO:0060089~molecular transducer activity  GO:0005125~cytokine activity  GO:0004872~receptor activity |
| IL6 | Down | -2.65 | 1.74E-02 | GO:0008083~growth factor activity  GO:0005125~cytokine activity |
| IL1A | Down | -7.64 | 3.78E-05 | GO:0070851~growth factor receptor binding  GO:0005125~cytokine activity |
| IL1B | Down | -8.68 | 1.59E-07 | GO:0044877~macromolecular complex binding  GO:0070851~growth factor receptor binding  GO:0005125~cytokine activity |
| NLRP3 | Down | -3.84 | 2.28E-03 | GO:0005539~glycosaminoglycan binding  GO:0042802~identical protein binding  GO:0042834~peptidoglycan binding |
| TREM2 | Up | 7.91 | 2.38E-07 | GO:0099600~transmembrane receptor activity  GO:0019207~kinase regulator activity  GO:0001530~lipopolysaccharide binding  GO:0060089~molecular transducer activity  GO:0004872~receptor activity |
| PTGS2 | Down | -9.38 | 3.16E-07 | GO:0046983~protein dimerization activity  GO:0042802~identical protein binding  GO:0019899~enzyme binding  GO:0016702~oxidoreductase activity |
